# Sodium valproate increases the brain isoform of glycogen phosphorylase: looking for a compensation mechanism in McArdle disease using a mouse primary skeletal-muscle culture *in vitro*

**DOI:** 10.1242/dmm.020230

**Published:** 2015-05-01

**Authors:** Noemí de Luna, Astrid Brull, Josep Maria Guiu, Alejandro Lucia, Miguel Angel Martin, Joaquin Arenas, Ramon Martí, Antoni L. Andreu, Tomàs Pinós

**Affiliations:** ^1^ Mitochondrial Pathology and Neuromuscular Disorders Laboratory, Vall d'Hebron Research Institute, Universitat Autònoma de Barcelona, Barcelona 08035, Spain; ^2^ Centro de Investigación Biomédica en Red de Enfermedades Raras (CIBERER), Madrid 28029, Spain; ^3^ Universidad Europea, Madrid 28670, Spain; ^4^ Instituto de Investigación ‘i+12’, Madrid 28041, Spain

**Keywords:** Glycogen phosphorylase, Glycogenolysis, McArdle disease, Myotubes, Sodium valproate

## Abstract

McArdle disease, also termed ‘glycogen storage disease type V’, is a disorder of skeletal muscle carbohydrate metabolism caused by inherited deficiency of the muscle-specific isoform of glycogen phosphorylase (GP-MM). It is an autosomic recessive disorder that is caused by mutations in the *PYGM* gene and typically presents with exercise intolerance, i.e. episodes of early exertional fatigue frequently accompanied by rhabdomyolysis and myoglobinuria. Muscle biopsies from affected individuals contain subsarcolemmal deposits of glycogen. Besides GP-MM, two other GP isoforms have been described: the liver (GP-LL) and brain (GP-BB) isoforms, which are encoded by the *PYGL* and *PYGB* genes, respectively; GP-BB is the main GP isoform found in human and rat foetal tissues, including the muscle, although its postnatal expression is dramatically reduced in the vast majority of differentiated tissues with the exception of brain and heart, where it remains as the major isoform. We developed a cell culture model from knock-in McArdle mice that mimics the glycogen accumulation and GP-MM deficiency observed in skeletal muscle from individuals with McArdle disease. We treated mouse primary skeletal muscle cultures *in vitro* with sodium valproate (VPA), a histone deacetylase inhibitor. After VPA treatment, myotubes expressed GP-BB and a dose-dependent decrease in glycogen accumulation was also observed. Thus, this *in vitro* model could be useful for high-throughput screening of new drugs to treat this disease. The immortalization of these primary skeletal muscle cultures could provide a never-ending source of cells for this experimental model. Furthermore, VPA could be considered as a gene-expression modulator, allowing compensatory expression of GP-BB and decreased glycogen accumulation in skeletal muscle of individuals with McArdle disease.

## Abstract (precis)

**Summary:** Use of this *in vitro* model showed that sodium valproate (VPA) can reverse the muscle phenotype from a McArdle-like to a normal histological and biochemical profile.

## INTRODUCTION

McArdle disease, also termed glycogen storage disease type V (OMIM^®^ number 232600), is a disorder of skeletal muscle carbohydrate metabolism caused by inherited deficiency of the skeletal-muscle isoform of glycogen phosphorylase (GP-MM). It is caused by pathogenic mutations in both copies of the GP-MM-encoding gene (*PYGM*), which is located in chromosome 11q12-11q13. Owing to their inability to use glycogen for fuelling muscle contractions, affected individuals commonly experience exercise intolerance, which typically consists of acute crises of early exertional fatigue, muscle stiffness and contractures, which, in the most severe cases, can be accompanied by rhabdomyolisis and subsequent myoglobinuria, thereby increasing the risk of renal damage (Lucia et al., 2008).

Although undetectable GP-MM activity is the common (and in fact diagnostic) observation in the differentiated cells (i.e. fibers) obtained from affected individuals' skeletal-muscle biopsies, cultured muscle cells derived from their muscle biopsies present GP activity (Martinuzzi et al., 1993; Meienhofer et al., 1977). Furthermore, human primary skeletal-muscle cultures obtained from biopsies of affected individuals do not differ from controls in that there is no excessive accumulation of periodic acid Schiff (PAS) staining material, and thus no abnormal glycogen deposits (Martinuzzi et al., 1993). However, other authors have failed to detect GP-MM in human primary skeletal-muscle cultures obtained from affected individuals or healthy controls (DiMauro et al., 1978; Sato et al., 1977).

Several types of treatments have been studied to reduce the symptoms in individuals with McArdle disease, with different and controversial results. No significant beneficial effects have been reported in patients receiving nutritional supplements such as branched-chain amino acids (MacLean et al., 1998), depot glucagon (Day and Mastaglia, 1985), dantrolene sodium (Poels et al., 1990), verapamil (Lane et al., 1984), vitamin B6 (Phoenix et al., 1998) [except in one recent case report (Sato et al., 2012)], high-dose oral D ribose (Steele et al., 1996) or high-dose creatine (Vorgerd et al., 2000). Low-dose creatine conferred a modest benefit on ischemic exercise in a few patients (Vorgerd et al., 2000). The ingestion of simple carbohydrates before engaging in strenuous exercise can alleviate their exercise-intolerance symptoms and diminish the risk of muscle rhabdomyolysis (Vissing and Haller, 2003), with supervised exercise training interventions also showing to be clinically beneficial (Haller et al., 2006; Maté-Muñoz et al., 2007; Santalla et al., 2014). The Cochrane review of pharmacological and nutritional treatment for McArdle disease includes the evidence from randomized controlled trials for improving exercise performance and quality of life in McArdle disease (Quinlivan et al., 2010).

**TRANSLATIONAL IMPACT**

**Clinical issue**

McArdle disease is a disorder of skeletal-muscle carbohydrate metabolism caused by inherited deficiency of muscle glycogen phosphorylase (GP-MM). This enzyme catalyzes and regulates the breakdown of glycogen into glucose-1-phosphate in muscle fibers. Thus, individuals with McArdle disease are unable to obtain energy from their muscle glycogen stores and, as such, present with exercise intolerance, typically manifested as acute crisis of undue, early exertional fatigue, muscle stiffness and contractures. In the more severe cases, these symptoms can be accompanied by rhabdomyolysis (breakdown of muscle fibers) and subsequent myoglobinuria (elevated urine levels of myoglobin, released as a muscle breakdown product). There are three *in vivo* animal models of the disease, two naturally occurring in Charolais calf and Merino sheep, respectively, and one experimentally generated in mice. The latter is a recently developed knock-in (KI) mouse model carrying the most common McArdle disease mutation (p.R50X) in the GP-MM-encoding gene (*Pygm*); these mice closely mimic the human disease phenotype. However, there is as yet no reported cellular model that mimics *in vitro* the main biochemical and histological alterations typically observed in biopsied muscle fibers from McArdle-affected individuals; that is, the absence of GP-MM and glycogen accumulation.

**Results**

The authors analyzed the expression of different GP isoforms [the brain (GP-BB) and liver (GP-LL) isoforms in addition to GP-MM] in cultured cells that were previously obtained from skeletal muscles of KI (GP-MM-deficient) or wild-type mice, at different stages of differentiation. They observed that GP-MM was the only GP isoform expressed *in vitro* in the differentiated cells obtained from wild-type mice, whereas GP-MM was not expressed in the differentiated cells from KI mice. In addition, only differentiated cells from KI mice accumulated glycogen in their cytoplasm, a characteristic (and, in fact, diagnostic) trait that occurs in the fibers of muscle biopsies from affected individuals. This *in vitro* model was used to assess whether sodium valproate (VPA) can reverse the muscle phenotype from a McArdle-like to a normal histological and biochemical profile. VPA activated the expression of only one GP isoform, GP-BB, in differentiated muscle cultures from KI mice, yet this isoform seemed to have beneficial functional consequences because it decreased intracellular glycogen accumulation.

**Implications and future directions**

These results demonstrate that primary skeletal-muscle cultures from McArdle KI mice represent a useful *in vitro* model that is able to replicate the biochemical and histological alterations associated with the human condition. In addition, the study shows that this model could be used as a high-throughput screening system for testing new drugs that aim to restore, at least partially, GP activity in the skeletal muscle tissue of individuals with McArdle disease, with potential therapeutic outcomes.

Because almost 50% of Caucasians with McArdle disease carry the nonsense p.R50X mutation, treatment with drugs that could potentially induce ‘read through’ of the generated premature termination codon could help to re-express GP-MM activity; however, gentamicin treatment failed to normalize phosphorus (^31^P) magnetic resonance [indicators of GP-MM activity in the skeletal muscles of individuals with McArdle disease (Schroers et al., 2006)]. Gene-therapy strategies have also been evaluated either *in vitro*, i.e. in human and sheep myoblast cultures deficient for GP-MM, or *in vivo*, i.e. in the ovine model of McArdle disease. Whereas, in myoblast cultures, GP-MM activity was restored after transfection with *PYGM* cDNA (Pari et al., 1999), intramuscular injection of adenovirus and adeno-associated vectors containing GP-MM expression cassettes in the ovine McArdle model only produced GP-MM functional activity in the surroundings of the injection site, and its expression diminished with time probably as a consequence of an immune response (Howell et al., 2008).

In addition to GP-MM, two other GP isoforms have been described: the liver and brain isoforms, which are encoded by the *PYGL* and *PYGB* genes, respectively. The brain isoform is the main GP isoform found in human and rat foetal tissues, including in the muscle, although its postnatal expression is dramatically reduced in the vast majority of tissues with the exception of the brain, where it remains as the major isoform. As described in the UCSC genome browser (http://genome-euro.ucsc.edu/index.html), both human, sheep and mouse *PYGB* genes present CpG islands in their promoters, and thus their expression might be regulated epigenetically through methylation of their promoters. In fact, postnatal downregulation of gene expression has been reported for many genes containing CpG promoters (Numata et al., 2012). Thus, any pharmacological treatment able to upregulate the expression of *PYGB* in the skeletal muscle of McArdle patients could theoretically alleviate the symptoms of the disease.

Valproic acid (VPA) is a short-chained fatty acid that has been used for many years in the treatment of epilepsy and bipolar disorders (McCoy et al., 1993; Sherard et al., 1980). Recent data suggest that this drug can modulate the epigenome by inhibiting histone deacetylases and activating the expression of methylated genes by stimulating active, replication-independent demethylation (Detich et al., 2003). The results of a recent study performed in a McArdle sheep model showed that enteral and intramuscular injection of VPA increased muscle expression of GP, although glycogen deposits and the expression of specific GP isoforms at the muscle tissue level were not analyzed (Howell et al., 2015).

We recently developed a p.R50X knock-in (KI) mouse model that presents with the main clinical features of the McArdle disease phenotype (Nogales-Gadea et al., 2012). Following on from this, in the present study we show that primary skeletal-muscle cultures derived from this murine model constitute a valid *in vitro* model to analyze and evaluate potential treatments for the disease because, in contrast to what occurs with muscle cultures derived from affected humans, these cells do not present GP activity and accumulate large amounts of glycogen deposits. Additionally, in these murine-derived muscle cultures, we observed how VPA treatment increased the expression of *Pygb*, providing an alternative mechanism that compensated, at least partly, for the lack of *Pygm* expression, as well as reducing polysaccharide accumulation.

## RESULTS

### Myotubes from primary skeletal-muscle cultures from homozygous p.R50X mice accumulate glycogen

**Fig. 1.**
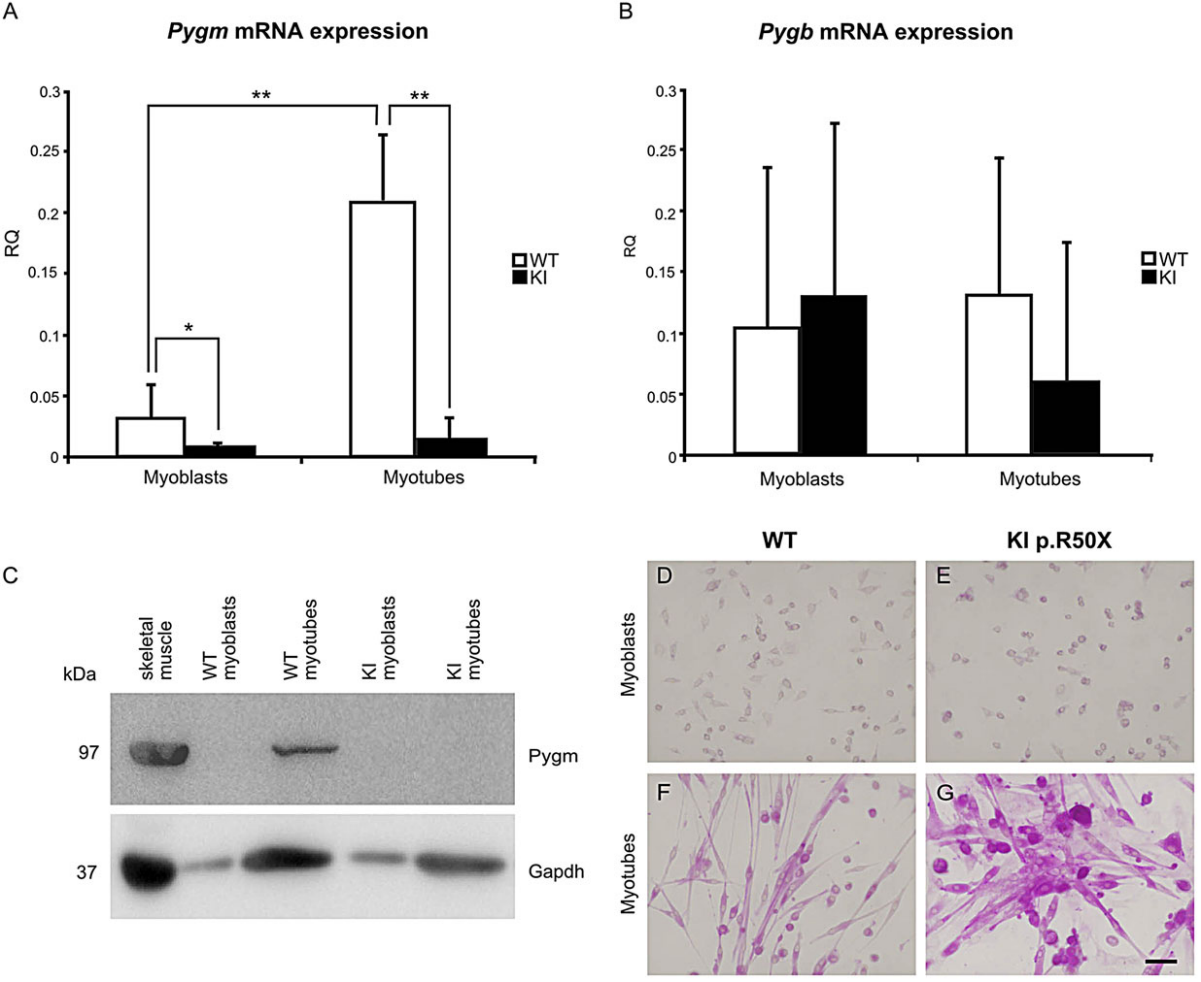
**Differential glycogen phosphorylase expression in mouse primary skeletal-muscle cultures.** Only WT myotubes expressed *Pygm* mRNA (A). Myoblasts and myotubes from WT and KI mice expressed *Pygb* mRNA with a high variation and no statistically significant differences between WT and KI (B). Presence of both *Pygm* transcript and protein (GP-MM) was observed only in WT myotubes (C). PAS staining of WT myoblasts (D), WT myotubes (F), KI myoblasts (E) and KI myotubes (G): only KI myotubes accumulated high glycogen levels (G). KI, knock-in; WT, wild type; RQ, relative quantification. *P<0.05 for the comparison KI versus WT, **P<0.001 for the comparison of KI versus WT. Scale bar: 50 µm.

We first analyzed *Pygm*, *Pygb* and *Pygl* expression at the undifferentiated (myoblasts) and differentiated (myotubes) stages of development in skeletal-muscle cultures derived from wild-type (WT) and KI McArdle mice. Myoblasts from WT and both myoblasts and myotubes from KI mice did not express *Pygm* mRNA (Fig. 1A). In WT muscle cultures, *Pygm* mRNA levels increased with muscle differentiation (*P*<0.001) (Fig. 1A). No significant differences were observed in *Pygb* mRNA levels among myoblasts and myotubes from WT and KI muscle cultures (Fig. 1B). Neither myoblasts nor myotubes from WT or KI mice expressed *Pygl* mRNA (data not shown). Western blot (WB) confirmed the mRNA results: GP-MM (*Pygm*) was only present in WT myotubes (Fig. 1C). GP-BB (*Pygb*) was not detected by western blot analysis in WT or KI myoblasts nor in WT or KI myotubes (data not shown).

We also performed a PAS staining in skeletal-muscle cultures from WT and KI mice. We observed that KI myotubes accumulated glycogen (Fig. 1G), whereas both WT and KI myoblasts and WT myotubes did not (Fig. 1D-F).

### Myotubes treated with VPA for 72 h expressed *Pygb* and reversed the glycogen accumulation

**Fig. 2.**
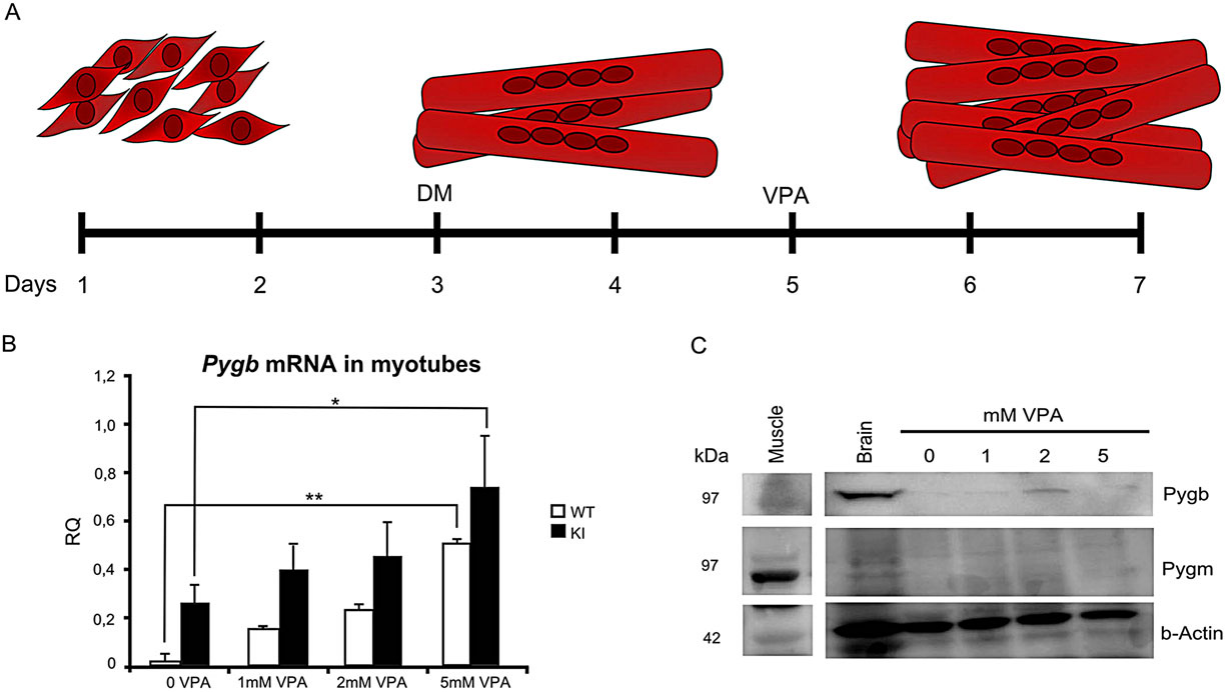
**VPA treatment increase *Pygb* expression in mice skeletal muscle cultures.** (A) After myotubes were formed, muscle cultures were treated with different concentrations of VPA for 72 h. (B) In both cell cultures (WT and KI), *Pygb* mRNA increased in treated cultures (**P<0.01, *P<0.05). (C) GP-BB (*Pygb*) protein was also detected with western blot analysis in WT and in KI cell cultures. Abbreviations: DM, Dulbecco's modified Eagle's medium; KI, knock-in; VPA, valproic acid; WT, wild type; RQ, relative quantification.

**Fig. 3.**
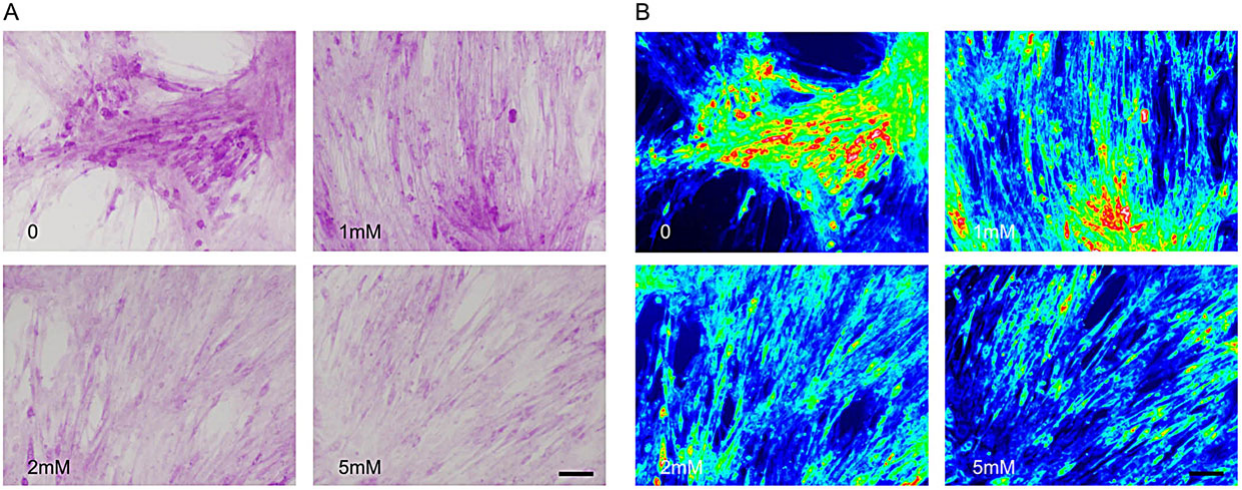
**Reduced glycogen accumulation in KI myotubes after treatment with VPA.** (A,B) We observed a gradual reduction in glycogen content as myotubes were treated with increasing VPA concentrations. (B) Red areas correspond to sites of major glycogen accumulation, whereas ‘cold’ colors (green, blue) represent sites of low PAS staining in myotubes. Scale bars: 50 µm.

WT and KI myoblasts and myotubes were treated for 72 h with VPA at 1, 2 and 5 mM. Myoblasts isolated from WT and KI mice and treated with VPA did not increase *Pygb* mRNA expression (data not shown). However, when VPA was added to the confluent myoblasts and differentiated myotubes, we observed an increased expression of *Pygb* mRNA, both in WT and in KI muscle cultures (*P*<0.01 and *P*<0.05, respectively). The highest amount of GP-BB (*Pygb*) protein expression was observed in myotubes treated with 2 mM VPA (Fig. 2). After 72 h of VPA treatment, no detachment of myotubes from the plate was observed, indicating that VPA was not toxic to the muscle culture (data not shown). The PAS staining on treated and non-treated myotubes showed a reduction in glycogen accumulation in cultures treated with 2 and 5 mM VPA (Fig. 3A). In the heat map in Fig. 3B, hot colors indicate high glycogen concentration, whereas cold colors such as green and blue show the zones with no glycogen accumulation.

**Fig. 4.**
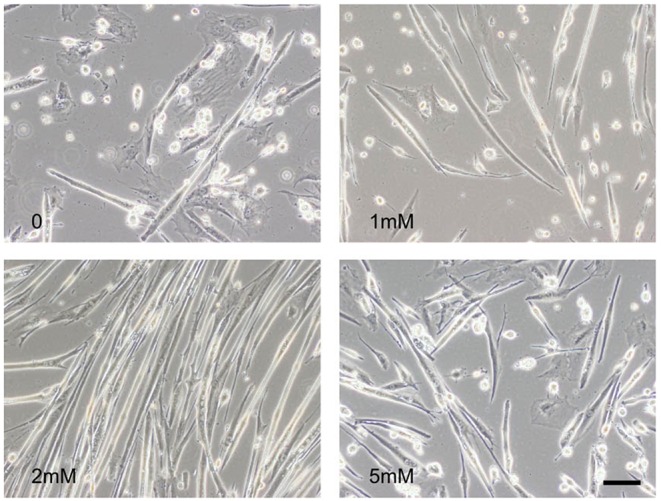
**Long-term VPA treatment of muscle cultures.** After 12 days of treatment, only cultures treated with 2 mM VPA showed healthy myotubes.

To test whether a longer period of VPA treatment could be toxic to the muscle cells, we changed the culture medium for each group (no VPA, or 1, 2 or 5 mM VPA) every 72 h over 12 days. After the 12-day period, the only muscle cultures that were maintained at a healthy myotube stage were muscle cultures treated with 2 mM of VPA (Fig. 4).

## DISCUSSION

The primary skeletal-muscle cultures derived from p.R50X KI mice represent a good cellular model of McArdle disease because they mimic the glycogen accumulation that is commonly found in the skeletal muscle from individuals with McArdle disease (Lucia et al., 2008). It has been previously reported that, in human culture cells either from affected or healthy individuals, *PYGM* expression contributed little to the total GP mRNA, whereas *PYGB* expression was predominant in myoblasts and *PYGB* and *PYGL* were both expressed in myotubes (Nogales-Gadea et al., 2010). Because *PYGB* and *PYGL* were the main GP isoforms expressed in human cultures, an increase in glycogen deposits was not observed in skeletal-muscle cultures derived from individuals with McArdle disease (Nogales-Gadea et al., 2010). By contrast, neither *Pygb* nor *Pygl* were expressed at any differentiation stage in our WT or KI mouse cultures, whereas GP-MM expression was restricted to WT myotubes, similarly to what has been shown to occur in the skeletal muscle of human adult healthy individuals. Additionally, vacuolization and increased intracellular PAS staining was also observed in KI mouse cultures. Thus, primary skeletal-muscle cultures derived from KI mice might represent a useful tool to test different pharmacological therapies prior to their evaluation in *in vivo* models.

A natural ovine model of McArdle disease has been described that is characterized by an adenine-for-guanine substitution at the intron 19 acceptor splice site of the *Pygm* gene (Tan et al., 1997). These sheep exhibit clinical features and morphological changes at the muscle tissue level that are similar to those shown by humans with the disease (Tan et al., 1997). In this animal model, regeneration of muscle fibers after necrosis induced by notexin injection reduced glycogen storage in regenerating muscle fibers, which showed re-expression of non-muscle (liver and brain) isoforms of GP, thereby indicating the need to investigate the potential functional benefit of inducing at the muscle tissue level these normally latent isoforms (Howell et al., 2014).

VPA exerts significant inhibitory effects on the activity of glycogen synthase (GS) kinase 3 beta (GSK3β) both *in vitro* and also *in vivo* (i.e. on endogenous GSK3β) (Chen et al., 2000). Because the inhibition of GSK3β might generate the accumulation of the more active unphosphorylated form of the GS enzyme, with its consequent increase in glycogen accumulation, we did not analyze GSK3β activity in VPA-treated or untreated KI mouse cells because, on the contrary, we observed a great reduction in glycogen accumulation *in vitro*, reaching normal levels. Additionally, it has also been observed that VPA has beneficial effects on skeletal-muscle myotubes, activating Akt signaling, stimulating gene transcription and protein synthesis, and promoting the survival of the cells via inhibition of apoptosis (Gurpur et al., 2009). We observed these ‘beneficial’ effects only when muscle cultures were treated with the 2 mM VPA concentration during a long period of time (12 days).

VPA induces histone acetylation of H3 histones, and is involved in the regulation of methylated genes by increasing the accessibility of the enzyme demethylase to the DNA (Detich et al., 2003; Milutinovic et al., 2006). The potential demethylation treatment of CpG islands of the *PYGB* promoter could allow the activation and transcription of the *PYGB* gene in skeletal muscle from individuals with McArdle disease as an approach to compensate for the lack of GP-MM. VPA treatment in humans could potentially have more effects on the glycogen accumulation because of the longer VPA half-life, compared with in mice (Loscher, 1978).

Our results demonstrate that mouse primary skeletal-muscle culture is a good study model of the disease because it mimics the phenotype of the muscle tissue from affected individuals. Furthermore, VPA can enhance *PYGB* expression *in vitro* and could be a candidate for the treatment of McArdle disease. Additional preclinical studies are needed to optimize the drug dosage to most effectively modulate *PYGB* gene expression.

## MATERIALS AND METHODS

All experimental procedures were approved by the Animal Care and Use Committee of the Vall d'Hebron Institut de Recerca (CEEA 35/04/08), and were in accordance with the European Convention for the Protection of Vertebrate Animals used for Experimental and Other Scientific Purposes (ETS 1 2 3) and the Spanish laws (32/2007 and R.D. 1201/2005).

### Mouse skeletal-muscle cultures

Myogenic precursor cells were isolated from 8-week-old KI McArdle mice carrying the p.R50X mutation in both copies of the *PYGM* gene (Nogales-Gadea et al., 2012) and from WT mice. All the muscles from lower limbs were dissected and digested in 0.2% pronase A (Calbiochem, Darmstadt, Germany) for 1 h at 37°C. After two washes with Dulbecco's modified Eagle's medium (D-MEM, Lonza Group Ltd., Basel, Switzerland), supplemented with 10% FBS (Lonza) and 100 units/ml penicillin (Lonza), 100 μg/ml streptomycin (Lonza) and 0.25 μg/ml amphotericin (Lonza) (PSF), cells were seeded in a non-coated 100-mm petri-dish in HAM-F10 media (Lonza) supplemented with 20% FBS, 2 mM glutamine (Lonza) and PSF for 1 h to allow fibroblasts to adhere. Thereafter, supernatants were plated in 2% gelatin-coated dishes containing HAM-F10 growth media supplemented with 20 ng/ml bFGF (Peprotech, Rocky Hill, NJ, USA). 2500 cells/cm^2^ were seeded in growth media to obtain myoblasts at day 7. To obtain myotubes, 12,500 cells/cm^2^ were seeded in growth media and, after 48 h, the culture media was replaced with one containing D-MEM, 5% horse serum (Lonza), 2 mM glutamine and PSF to allow myoblasts to fuse and to form myotubes. Myotubes were analyzed after 5 days in differentiation medium. The medium was changed twice a week, and the muscle cultures were examined to confirm confluent growth of myoblasts and myotubes. Each condition was performed in triplicate.

### VPA treatment in cell cultures

After 48 h in differentiation media in which myotubes were formed, we added VPA (Sigma-Aldrich, Madrid, Spain) at different concentrations (1, 2 and 5 mM) during an additional 72-h period.

### RNA extraction and real-time polymerase-chain reaction (PCR)

Total RNA from mouse skeletal muscle cultures treated or not with VPA was extracted using Trizol (Life Technologies, Madrid, Spain). 0.5 μg of total RNA was DNAse-treated (Life Technologies, Madrid, Spain) and was then reverse-transcribed into cDNA using the high capacity cDNA RT kit (Life Technologies, Madrid, Spain).

Quantification of *Pygm*, *Pygb*, *Pygl* and *Gapdh* (used as an internal standard) transcripts was performed using TaqMan Universal Master Mix technology (Life Technologies). Quantitative PCR was performed in a total reaction volume of 20 μl per well. The primers used for real-time PCR were designed by Applied Biosystems (Roche Molecular Systems) (*Pygm* Mm 00478582_m1, *Pygb* Mm 00464080_m1, *Pygl* Mm 01289790_m1 and mouse *Gapdh* endogenous control). The comparative CT method (ΔΔCT) for relative quantification of gene expression was used. The Student's *t*-test was used for statistical comparisons between the data obtained in KI versus WT mice.

### Western blot

Cell and muscle samples corresponding to each experimental condition were trypsinized from the culture dish and homogenized with a lysis buffer containing 40 mM glycophosphate, 40 mM NaF, 10 mM EDTA and 20 mM of β-mercaptoethanol (final pH=6). The samples were placed in boiling water for 3 min and centrifuged at 9500 ***g*** for 3 min, before 100 μg of protein was applied to each lane. Unspecific binding sites on the blots were blocked by incubation in 5% low-fat dried milk powder in a phosphate buffered saline. Thereafter, primary rabbit polyclonal antibody anti-PYGB (kindly provided by Dr K. Nowak, Harry Perkins Institute of Medical Research, Nedlands, WA, Australia), the primary goat anti-PYGM [kindly provided by Dr Martinuzzi, Istituto di Ricovero e Cura a Carattere Scientifico Eugenio Medea – Associazione ‘La Nostra Famiglia', Conegliano (Treviso), Italy], mouse anti-GAPDH (Ambion, Life Technologies) or anti-β-actin (Sigma-Aldrich) were added. Peroxidase-conjugated anti-rabbit (Jackson InmunoResearch, West Grove, PA, USA), peroxidase-conjugated anti-goat (Santa Cruz Biotechnology Inc., Heidelberg, Germany) and peroxidase-conjugated anti-mouse (Dako, Glostrup, Denmark) secondary antibodies were applied when using anti-PYGB antibody, anti-PYGM antibody and anti-GAPDH and anti-β-actin, respectively.

### Periodic acid Schiff (PAS) staining in cultured cells

Both cell cultures treated or not treated with VPA were incubated with 1% of periodic acid (Sigma-Aldrich) in acetic acid for 30 min. Cells were washed in 0.1% sodium metabisulfite (Sigma) in 1 mM hydrochloric acid. Thereafter, they were incubated in a Schiff solution (Merck, Darmstadt, Germany) for 15 min after 0.1% sodium metabisulfite in 1 mM hydrochloric acid wash, and cells were observed in an inverted Olympus FSX100 microscope.
